# Curcumin Mitigates Malathion-Induced Renal Injury: Suppression of Apoptosis and Modulation of NF-κβ/TNF-α and Nrf2, and HO-1 Signaling

**DOI:** 10.3390/metabo13111117

**Published:** 2023-10-30

**Authors:** Mamdouh Eldesoqui, Magda E. Ahmed, Mona A. Abdel-Kareem, Mohamed Moharram Badawy, Amal Fahmy Dawood, Abdelaty Shawky Mohamed, Ateya Megahed Ibrahim, Ahmed A. El-Mansi, Mohamad El-Sherbiny, Mahmoud Hendawy

**Affiliations:** 1 Department of Human Anatomy and Embryology, Faculty of Medicine, Mansoura University, Mansoura 35516, Egypt; mamrah@um.edu.sa (M.E.); magi86@mans.edu.eg (M.E.A.); hendawy016@mans.edu.eg (M.H.); 2 Department of Basic Medical Sciences, College of Medicine, AlMaarefa University, P.O. Box 71666, Riyadh 11597, Saudi Arabia; ashawky@um.edu.sa; 3 Department of Anatomy and Embryology, Faculty of Medicine, Kafrelsheikh University, Kafrelsheikh P.O. Box 33516, Egypt; mona_abdelkarim2014@med.kfs.edu.eg; 4 Forensic Medicine and Clinical Toxicology Department, Faculty of Medicine, Mansoura University, Mansoura 35516, Egypt; dr_mmuharram85@mans.edu.eg; 5 Forensic Medicine and Clinical Toxicology Department, Faculty of Medicine, Delta University for Science and Technology, Gamasa 11152, Egypt; 6 Department of Basic Medical Sciences, College of Medicine, Princess Nourah bint Abdulrahman University, P.O. Box 84428, Riyadh 11671, Saudi Arabia; afdawood@pnu.edu.sa; 7 Pathology Department, Faculty of Medicine, Mansoura University, Mansoura 35516, Egypt; 8 Department of Nursing, College of Applied Medical Sciences, Prince Sattam bin Abdulaziz University, Al-Kharj 11942, Saudi Arabia; a.eleglany@psau.edu.sa; 9 Department of Family and Community Health Nursing, Faculty of Nursing, Port Said University, Port Said P.O. Box 42511, Egypt; 10 Biology Department, College of Science, King Khalid University, Abha 61413, Saudi Arabia; aelmansi@kku.edu.sa

**Keywords:** curcumin, malathion, renal toxicity, apoptosis, antioxidant

## Abstract

Malathion is one of the most used organophosphorus pesticides that is used for many reasons such as agriculture and industry. Human exposure to malathion may occur through various means, such as eating food that has been treated with it. Malathion not only increases oxidative stress but also decreases the antioxidant capacity. Curcumin is a powerful antioxidant with many pharmacological actions. Curcumin can act as a free radical scavenger and inhibit the activation and nuclear translocation of NF-κB. Curcumin could combat the lipid peroxidation and antioxidant depletion that trigger the apoptotic pathways. This study aims to examine the antioxidant, anti-inflammatory, and antiapoptotic effects of curcumin. Twenty-four Sprague Dawley rats were divided into four groups (six rats each): control, curcumin, malathion, and malathion + curcumin groups. At the assigned time, blood samples were used for the assessment of serum creatinine, and the kidneys were excised and washed; parts of them were used for the assessment of total oxidant status (TOS), oxidative stress index (OSI), the oxidative stress marker malondialdehyde (MDA), total antioxidant capacity (TAC), and glutathione (GSH) activity, other parts were fixed in formalin for further staining. Histopathological evaluation was performed for the fixed specimens after staining with H&E, sirus red, and the immunohistochemical staining for NF-κβ, TNF-α, Caspase-3, Nrf2, and HO-1. Curcumin significantly decreases the serum creatinine after malathion exposure and significantly restores the oxidant/antioxidant balance by increasing TAC and GSH and decreasing TOS, OSI, and MDA. Curcumin exerts its reno-protective effect and restores the histological architecture of the kidney by downregulating the immune expression of NF-κβ, TNF-α, and Caspase-3 and upregulating the expression of Nrf2 and HO-1. This study concluded that curcumin protects against nephrotoxicity caused by malathion by exerting its antioxidant, anti-inflammatory, and anti-apoptotic capabilities.

## 1. Introduction

Extensive consumption of organophosphorus pesticides in various areas such as crop growing and industry can initiate a lot of disorders for people and the environment [1]. One of the most used organophosphate pesticides is malathion. It is frequently applied to get rid of ectoparasites and home pests, keep grain from spoiling, and kill arthropods causing diseases [2]. 

Humans can be exposed to malathion through various means, including consumption of food that has been treated with pesticide, as well as ingestion of water that has been contaminated. In the nervous system, malathion stops the action of the enzyme cholinesterase at synapses, causing an increase in acetylcholine. It causes the same effect in all living organisms like animals [3].

Due to their lipophilic nature, and quick and easy intestinal absorption, pesticides like malathion can penetrate all tissues and cause a variety of clinical disorders including kidney damage [4]. The liver and kidney are among the primary affected organs of malathion toxicity, which is mediated by oxidative stress initiated by ROS [5], and higher levels of ROS result in tissue damage [1]. Pasaoglu and his colleagues reported that organophosphorus pesticides caused an increased production of reactive oxygen species (ROS) that induce oxidative stress, moreover, it attenuates the antioxidant defense capacity [6]. Yonar et al. (2017) reported that curcumin restored the activities of key antioxidants, including superoxide dismutase, catalase, glutathione peroxidase, and glutathione-S-transferase [7]. Al-Othman et al. (2011) reported elevated malondialdehyde (MDA) levels and attenuated antioxidant activity and glutathione levels in malathion-treated rats [8]. 

Nrf2 and NF-β signaling pathways collaborate to sustain the physiological equilibrium of cellular redox status and control how cells react to stress and inflammation [9]. Genes that affect inflammation and the response to oxidative stress are regulated by NF-kB. As a result, it controls cellular damage and inflammatory reactions [10]. In addition to maintaining homeostasis and redox equilibrium in cells and tissues, Nrf2/ARE signaling is essential for the defense against oxidative stress [11].

Curcumin is a powerful antioxidant in both food and biological systems and shows many pharmacological actions and therapeutic effects [12]. Curcumin and similar substances can decrease the production of free radicals, function as free radical scavengers and antioxidants, stimulate glutathione-S-transferase, and prevent lipid peroxidation. Curcumin, as an antioxidant, would protect the kidney exposed to malathion by raising its antioxidant status because of the significant role of ROS in renal toxicity [13,14].

Therefore, the main goal of this work was to highlight the importance of curcumin as an antioxidant, anti-inflammatory, and antiapoptotic in improving biochemical and histopathologic complications following exposure of rats to malathion.

## 2. Materials and Methods

### 2.1. Chemicals

Malathion (57% commercial grade) is available in a white liquid formula, obtained from Al Nasr Company (Cairo, Egypt) for chemical industries, Egypt, and the remaining chemicals and kits—if not mentioned—were acquired from Sigma-Aldrich Company. The essential dose of malathion and curcumin were liquefied in distilled water before oral administration.

### 2.2. Animals and Experimental Design

Twenty-four adult male albino rats weighing 200–220 g were used. The animals were housed in metallic cages (three rats/cage) with softwood chips for bedding. The rats were kept at a constant temperature (20 °C), humidity (50%), and on a fixed (12 h–12 h) dark/light cycle. They had free access to a standard diet and drinking water. The doses of curcumin were chosen in accordance with the previously standardized values, while the dose of malathion was chosen to equal 1/20 of the LD50. After one week of acclimatization, rats were divided into four groups (six rats each). The study groups were:

1. Group 1 (Control group): untreated and served as control;
2. Group 2 (curcumin group): received curcumin solution by oral gavage at a dose of 150 mg/kg/day [15,16];
3. Group 3 (malathion group): received malathion by oral gavage at a dose of 100 mg/kg/day [17];
4. Group 4 (curcumin + malathion group): received curcumin solution by oral gavage at a dose of 150 mg/kg/day [15,16] and, three hours later, received malathion at the same dose applied to group 3 (i.e., 100 mg/kg/day) [17].

After 4 weeks of drug administration, ketamine (50 mg/kg) was used to anesthetize fasted rats, and 5 mL of venous blood was taken for hematobiochemical investigation. After the withdrawal of blood, all rats were sacrificed, and both kidneys were freshly excised and washed with cold 0.9% NaCl solution for preparation of histopathologist evaluation.

### 2.3. Creatinine Level Assessment

To separate serum, the collected blood samples were centrifuged for 15 min at 9000× *g*, and the separated sera were used for creatinine levels using a commercially available Creatinine Assay Kit, according to the manufacturer’s instructions.

### 2.4. Assessment of Tubular Kidney Injury Molecule (Kim-1)

Tubular Kidney injury molecule (Kim-1) was measured using Rat Kim-1 Sandwich ELISA Kit according to the manufacturer’s instructions (Rat Kim-1 ELISA Kit, Boster immuneboster; Fremont, CA, USA).

### 2.5. Assessment of Oxidant and Antioxidant State

#### 2.5.1. Assessment of Malondialdehyde (MDA) and Antioxidant Enzymes (GSH)

Parts of the kidneys were homogenized (10% *w*/*v*) in a pH 7.4 0.1 M Tris-HCl buffer. At 4 °C, the homogenates were centrifuged for 10 min at 3000 rpm. The kidney homogenate’s subsequent supernatant is included in the measurement of oxidative stress markers Malondialdehyde (MDA) and reduced glutathione (GSH), which were detected by the relevant kits (Biodiagnostic kits, Bio-Diagnostics, Dokki, Giza, Egypt).

#### 2.5.2. Assessment of Total Oxidant Status (TOS) 

The levels of TOS were estimated in the kidney homogenates using Erel’s method, which depends on the oxidation of ferrous ion to ferric ion indicators in the presence of various oxidative species in the reaction mixture. Then, the produced ferric ion complex—with xylenol orange in the medium to produce a colored complex with such intensity that it measures spectrophotometrically—was analyzed and the results obtained are expressed in terms of micromole hydrogen peroxide equivalent per liter (μmol H_2_O_2_ equivalent/L).

#### 2.5.3. Measurement of Total Antioxidant Capacity (TAC)

Total antioxidant capacity (TAC) was estimated in the kidneys using Colorimetric Assay Kit (Catalog #K274-100; BioVision Incorporated, Milpitas, CA, USA). The antioxidant equivalent concentrations were measured at 570 nm as a function of Trolox concentration, according to the manufacturer’s instructions.

#### 2.5.4. Measurement of Oxidative Stress Index (OSI)

Oxidative stress index (OSI) was estimated for all control, treated, and non-treated groups. OSI was determined by the TOS: TAC ratio, which represents the oxidative stress index (OSI) arbitrary unit. 

### 2.6. Histopathological Examination

The kidney tissues were fixed in 10% formalin and embedded in paraffin. For light microscopic analysis, 3–5 µm tissue sections were cut from the paraffin blocks and stained with hematoxylin, eosin, and Sirius red. Using an Olympus light microscope (Olympus BX51, Tokyo, Japan) and an accompanying camera (Olympus E-330, Olympus Optical Co. Ltd., Tokyo, Japan), the sections were examined and photographed.

### 2.7. Immunohistochemical Staining for Detection of NFκβ, TNF-α, Nrf2, Ho-1, and Caspase-3

Sections were deparaffinized and endogenous peroxidases were inhibited with 3% H_2_O_2_ at room temperature. Antigenic sites were activated by treating the sections with 10 mM sodium citrate buffer (pH = 6) and heating for 10 min at 95°C, then left to cool for 30 min. Non-specific binding was blocked with 5% bovine serum albumin, then sections were then treated overnight with a primary antibody at 4 °C against:

1. NFκβ: ABclonal Catalog No. A3108, Polyclonal Antibody in a dilution of 1/100;
2. TNF-α: Servicebio Catalog No. GB11188, Polyclonal Antibody in a dilution of 1/1000;
3. Caspase-3: Servicebio Catalog No. GB11532, Polyclonal Antibody in a dilution of 1/1000;
4. Nrf2: Servicebio Catalog No. GB113808, Polyclonal Antibody in a dilution of 1/1000;
5. Ho-1: ABclonal Catalog No. A19062, Polyclonal Antibody in a dilution of 1/100.

The tissue sections were exposed to Diaminobenzidine (DAB) reagent to highlight peroxidase activity. Subsequently, the sections were incubated in PBS at 4°C overnight. Slides were treated with universal secondary antibodies (anti-mouse IgG or anti-rabbit IgG) from the Mouse/Rabbit poly detector plus the DAB HPR brown detection system (USA) for 30 min. Afterward, the slides were rinsed in Phosphate-buffered saline (PBS) to locate the binding sites of the primary antibody. DAB was applied for 4 min, followed by counterstaining the sections with hematoxylin. In the negative control group, PBS was substituted for the primary antibody. Lastly, slices were dried, fixed, and examined.

### 2.8. Measurement of the Area Percentage of Ho-1, TNF-α, Caspase-3, Nrf 2, and NFKB Positive Reactions in the Kidney

The immunoreactive area percentage in the immunoassayed sections was analyzed using Image J software (v 1.53, National Institutes of Health, USA. https://imagej.nih.gov/ij/ (accessed on 03 October 2023)) using the color deconvolution plugin and the H-DAB vector.

### 2.9. Statistical Analysis and Data Interpretation

Collected data were recorded and evaluated with IBM SPSS Statistics for Windows, Version 22.0 (IBM Corp., Armonk, NY, USA) and GraphPad Prism version 9.0.0 (121). Qualitative data were defined by means of numbers and percentages. Quantitative data were designated using mean and standard deviation for parametric data after analysis of normality using the Shapiro–Wilk test. One-way ANOVA test was used to compare more than 2 independent groups using the post hoc Tukey’s test to distinguish pair-wise assessment. The statistical significance of the attained results was defined at the (0.05) level.

## 3. Results

There were no reported toxic manifestations or mortality of rats during the experimental period.

### 3.1. Effect of Curcumin on Serum Creatinine after Malathion Intoxication

As shown in Table 1, the findings of the current research revealed that serum concentrations of creatinine were significantly increased in malathion rats (group 3) in comparison with the control group and rats treated with combined curcumin and malathion (group 4). There was a statistically significant improvement (reduction) in serum concentrations of creatinine in rats treated with the combined curcumin and malathion (group 4) as compared to rats exposed to malathion alone.

### 3.2. Effects of Curcumin Administration on the Cellular Oxidative and Antioxidant Status

The levels of TOS, OSI, TAC, MDA, and the activities of GSH were estimated in the homogenate of kidney tissues of control, curcumin-treated, and non-treated malathion-intoxicated rats (Table 2). The results showed that the levels of cellular TOS, OSI, and MDA, as oxidative markers, were significantly increased with a reduction in the activities of TAC and GSH in rats intoxicated with malathion (group 3) compared to control rats (group 1) (Table 2); moreover, a protective antioxidant activity of curcumin was reported when healthy control rats received curcumin at a dose of 150 mg/kg/day (group 2). In addition, malathion-intoxicated rats treated with curcumin (group 4) showed a significant improvement in the cellular antioxidant status compared to non-treated intoxicated rats (group 3) (Table 2). There was a significant reduction in the levels of TOS, OSI, and MDA with an increase in the antioxidant activity parameters—TAC, and GSH enzyme—signifying that the potential activity of curcumin might proceed via antioxidant pathways.

### 3.3. Effect of Curcumin on the Kidney Structure after Malathion Intoxication

**Figure 1:**
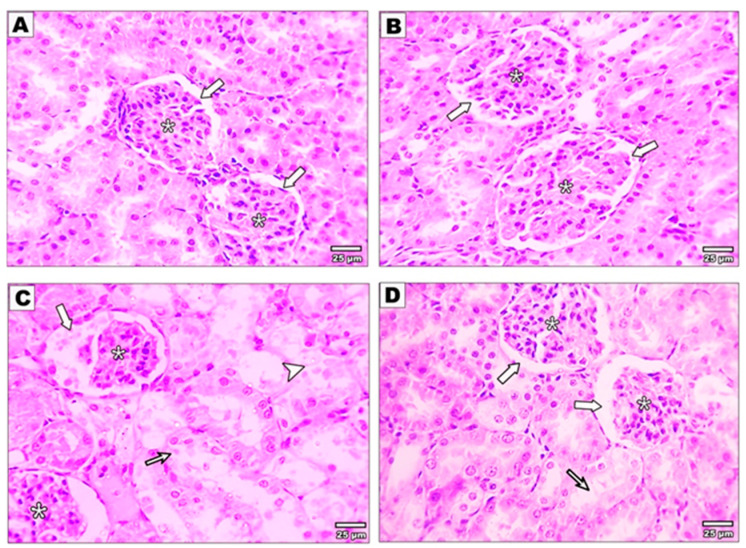
Photomicrographs of the kidney sections stained with H&E: (**A**) The control and (**B**) curcumin groups showed normal glomeruli (*) surrounded by Bowman’s capsule formed of two layers separated by Bowman’s space (thick arrow) surrounded with normal tubular architecture. On the other hand, the malathion group (**C**) shows shrunken glomerular tufts (*) and dilated Bowman’s capsule with eosinophilic proteinaceous material, severe tubular hydropic degeneration (thin arrow), and coagulative necrosis (arrowhead), particularly in corticomedullary and medullary zones. Malathion + curcumin group (**D**) shows normal glomeruli (*), Bowman’s capsule (thick arrow), and mild tubular hydropic degeneration (thin arrow).

Sections stained with Hematoxylin and Eosin of the control rats and curcumin-treated rats revealed the typical structure of the kidney. The histological examination of the renal cortex exhibited proximal convoluted tubules with narrow lumen, cuboidal cell lining, and acidophilic granular cytoplasm. In contrast, distal convoluted tubules had thin walls, wide lumen, and less intense staining than the proximal tubules. The glomeruli were surrounded by Bowman’s capsule composed of two layers divided by Bowman’s space (Figure 1A,B). Malathion-intoxicated rats revealed distorted architecture compared with the control, curcumin, and malathion + curcumin groups. The glomeruli showed a dilated Bowman’s capsule with eosinophilic proteinaceous material and a shrunken glomerular tuft. There were mononuclear cell infiltrations and dilated congested blood vessels in the interstitium. Furthermost of the tubules revealed hydropic degeneration, wide lumen, damaged lining epithelium, and vacuolated cytoplasm. Hyaline casts were found inside some tubules (Figure 1C). However, the kidneys of malathion + curcumin-treated rats (group 4) showed more re-establishment of the typical structure compared with the malathion group. Some glomeruli appeared normal with normal Bowman’s capsules, and others showed dilated capsules. Most tubules appeared normal. There was minimal interstitial tissue inflammation with mild hydropic degeneration in some tubules (Figure 1D). 

### 3.4. Effect of Curcumin on the Fibrotic Changes after Malathion Intoxication

**Figure 2:**
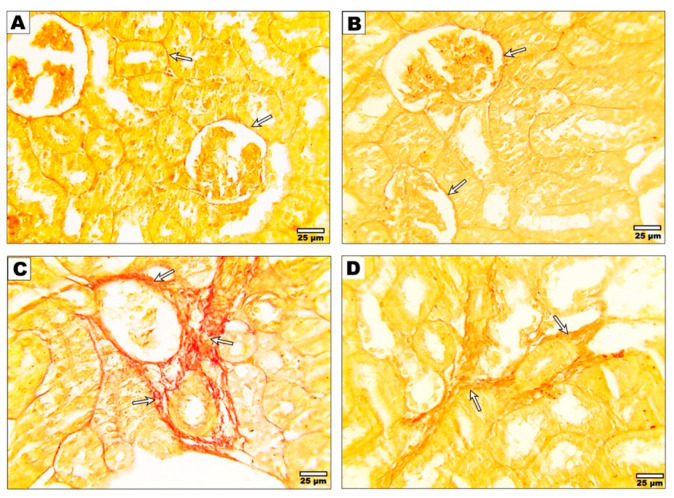
Photomicrographs of sections stained with Sirus red from kidneys of control (**A**) and curcumin (**B**) rats showed thin collagen fibers observed around glomeruli, renal tubules, and interstitial tissue (arrows), while malathion-treated rats (**C**) showed a relative increase in the thickness of collagen fibers around glomeruli, interstitial tissue, and renal tubules (arrows). Rats who received malathion + curcumin (**D**) revealed thinner collagen fibers around tubules and in the interstitium (arrows).

Regarding the Sirus red staining, the kidneys of control and curcumin-treated rats exhibited the presence of thin collagen fibers surrounding the glomeruli, renal tubules (Figure 2A,B), and interstitial tissue; in contrast, malathion-intoxicated rats (group 3) displayed thicker collagen fibers compared to the control group. Conversely (Figure 2C), rats treated with malathion + curcumin (group 4) presented thinner collagen fibers around the tubules and in the interstitium when compared to the group that received only malathion (Figure 2D).

### 3.5. Morphometric Results of the Immunohistochemical Staining

NF K-β, TNF-α, Nfr2, Ho-1, and Caspase- 3-stained slides from the kidney were submitted for morphometric image analysis. 

**Figure 3:**
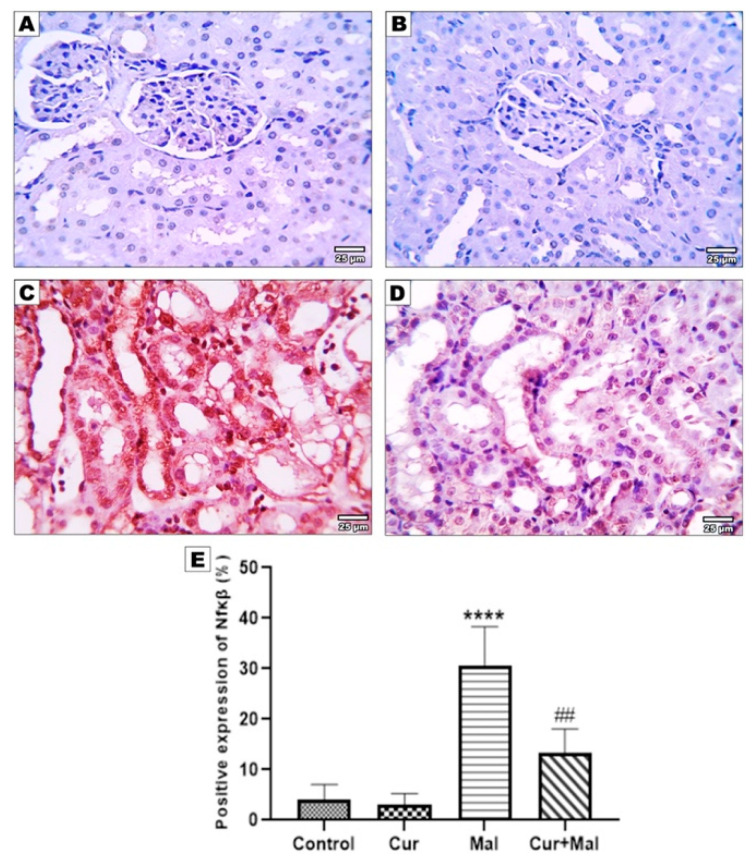
Immunohistochemical expression of NF- kβ in different groups: control (**A**), curcumin (**B**), malathion (**C**), and malathion + curcumin (**D**); (**E**) represents the morphometric analysis of the immunoreactive area. The data are represented as mean ± SD, and the data were compared using ANOVA and post hoc Tukey’s test. A significant difference was considered if ρ-value < 0.05. **** Significance versus the control group and ## significance versus malathion group.

**Figure 4:**
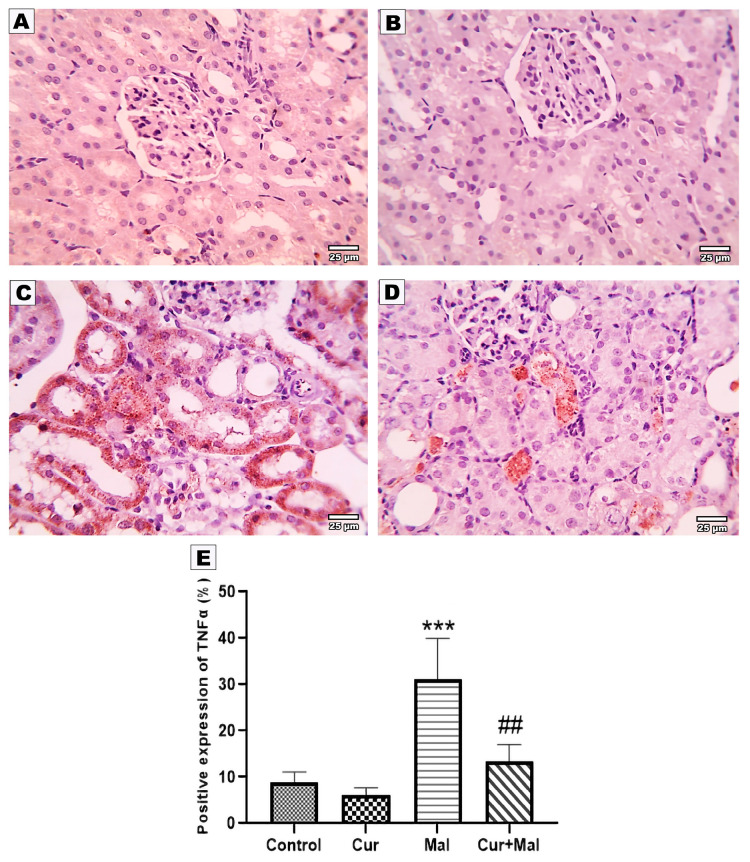
Immunohistochemical expression of TNF-α in the different groups: control (**A**), curcumin (**B**), malathion (**C**), and malathion + curcumin (**D**); (**E**) represents the morphometric analysis of the immunoreactive area. The data are represented as mean ± SD, and the data were compared using ANOVA and post hoc Tukey’s test. A significant difference was considered if ρ-value < 0.05. *** Significance versus the control group and ## significance versus malathion group.

**Figure 5:**
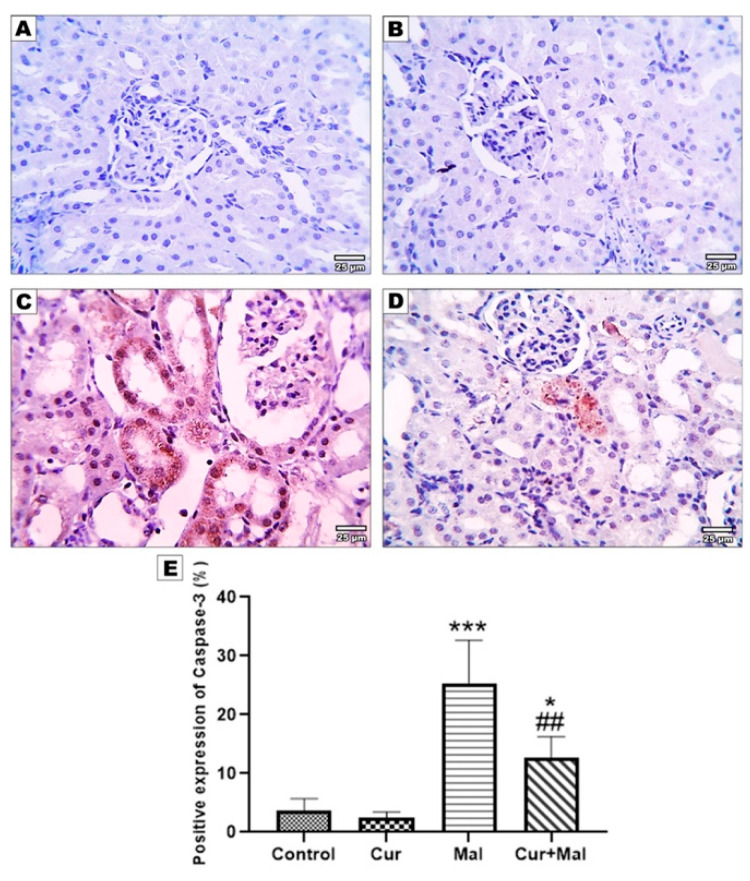
Immunohistochemical expression of Caspase-3 in the different groups: control (**A**), curcumin (**B**), malathion (**C**), and malathion + curcumin (**D**) groups; (**E**) represents the morphometric analysis of the immunoreactive area, The data are represented as mean ± SD, and the data were compared using ANOVA and post hoc Tukey’s test. A significant difference was considered if ρ-value < 0.05. *** Significance versus the control group and * ## significance versus malathion group.

Compared with the control group, malathion-receiving rats (group 3) showed a significantly increased area% of positive NF K-β, TNF-α, and Caspase- 3 immunoreactivities (*p* > 0.0001, *p* = 0.0001, and *p* = 0.0001, respectively); however, compared with the malathion group, malathion- and curcumin-receiving rats (group 4) showed a significantly decreased area% of positive NF K-β, TNF-α, and Caspase- 3 immunoreactivities (*p =* 0.0013, *p =* 0.0008, and *p =* 0.0056, respectively). On the other hand, there was no significant difference in NF K-β or TNF-α when compared with the control group but there was significant elevation in Caspase- 3 (*p =* 0.0451) (Figure 3, Figure 4 and Figure 5).

**Figure 6:**
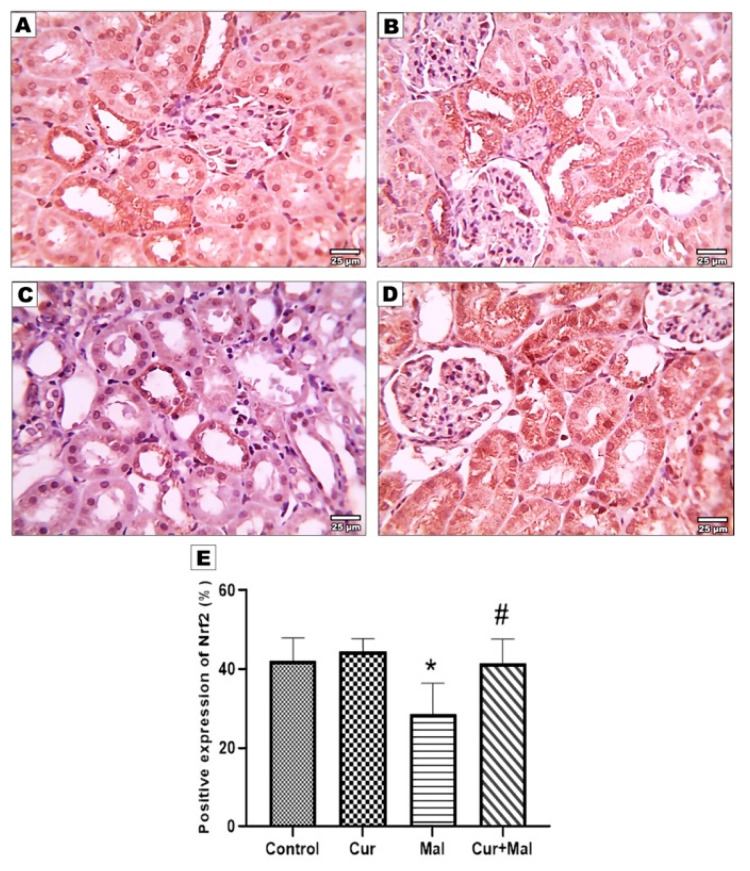
Immunohistochemical expression of Nrf2 in the different groups: control (**A**), curcumin (**B**), malathion (**C**), and malathion + curcumin (**D**) groups; (**E**) represents the morphometric analysis of the immunoreactive area. The data are represented as mean ± SD, and the data were compared using ANOVA and post hoc Tukey’s test. A significant difference was considered if ρ-value < 0.05. * Significance versus the control group and # significance versus malathion group. Compared with the control group, malathion-receiving rats showed a significantly decreased area% of positive Nrf2 and Ho-1 immunoreactivity (*p* = 0.0338 and *p =* 0.0047, respectively); however, compared with the malathion group, malathion and curcumin-receiving rats (group 4) showed a significantly increased area% of positive Nrf2 and Ho-1 immunoreactivity (*p* = 0.0438 and *p =* 0.0139, respectively) (Figure 6 and Figure 7).

**Figure 7:**
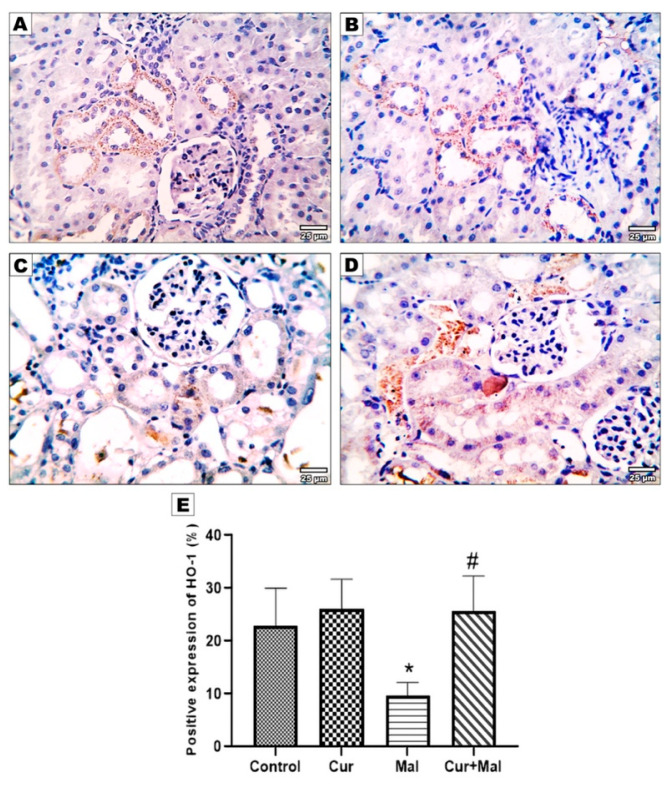
Immunohistochemical expression of HO-1 in the different groups: control (**A**), curcumin (**B**), malathion (**C**), and malathion + curcumin (**D**) groups; (**E**) represents the morphometric analysis of the immunoreactive area. The data are represented as mean ± SD, and the data were compared using ANOVA and post hoc Tukey’s test. A significant difference was considered if *p*-value < 0.05. * Significance versus the control group and # significance versus malathion group.

Compared with the control group, malathion-received rats showed a significantly decreased area% of positive Nrf2 & Ho-1 immunoreactivity (*p* = 0.0338 and *p* = 0.0047 respectively). However, compared with the malathion group, malathion & curcumin-received rats (group 4) showed a significantly increased area% of positive Nrf2 & Ho-1 immunoreactivity (*p* = 0.0438 & *p* = 0.0139, respectively) (Figure 6 and Figure 7).

## 4. Discussion

In developing countries, poisoning by pesticides has emerged as a significant global health issue. Pesticide overuse and misuse result in serious poisoning and millions of deaths [18]. Malathion is one of the most common globally utilized organophosphorus pesticides [19]. 

Possamai and his colleagues reported that the kidneys, lungs, diaphragm, and liver were the most susceptible organs to oxidative damage following acute management with malathion [20]. According to El-Baz et al. (2016), curcumin possesses powerful antioxidant and preventative activities against oxidative stress [19]. That is why the current study was designed to emphasize the function of curcumin as an antioxidant in mitigating or eliminating the molecular and histopathological problems resulting from intoxication of the rats with malathion. Curcumin has been shown in a variety of preclinical studies to have therapeutic benefits, including anti-inflammatory, anti-angiogenic, antioxidant, wound healing, anti-proliferative, sensitizer to exert synergistic anticancer activity, and anti-carcinogenic efficacy [21,22].

Concerning biochemical analysis, the current study demonstrated that malathion-exposed rats were associated with a significant increase in creatinine levels in comparison to the control group as well as to rats treated with combined curcumin and malathion. This seems that curcumin has protective actions for malathion-exposed rats. Kata (2020) has demonstrated that mice who received an intraperitoneal injection of the pesticide malathion revealed significantly higher serum creatinine concentrations than mice in the control group (*p* 0.05); furthermore, this increase in serum creatinine was associated with histological alteration in renal structure. Thus, he concluded that six days of exposure to malathion can cause kidney damage in mice [23].

Notably, free radicals and oxidative stress working as oxidants to damage or counteract the effect of the antioxidant system were the primary causes of malathion-induced toxicity [24]; however, renal tubular insufficiency, inadequate glomerular filtration, and kidney injury resulting from pesticide exposure were the main causes of the rise in creatinine [25]. 

The generation of ROS is typically regulated under normal physiological conditions. Cellular oxidative stress happens when there is a disruption to the balance between the production of ROS and the antioxidant capacity of the cells [26].

The oxidative stress is created by malathion biotransformation into malaoxon and via malathion detoxification through conjugation with glutathione, which leads to the generation of ROS and depletion of antioxidant markers, as evidenced by the formation of MDA, and depletion of GSH [27]. 

The mean renal TOS, OSI, and MDA were significantly higher in rats that received malathion than in the control group; however, in rats treated with combined curcumin and malathion, mean kidney tissue TOS, OSI, and MDA were significantly reduced compared with rats exposed to malathion (group 3), indicating the malathion-mediated oxidative stress. 

As regards the renal antioxidant status, the mean kidney tissue TAC and GSH were significantly reduced in rats exposed to malathion compared with the control group; this reduction was significantly ameliorated in rats treated with combined curcumin and malathion compared with rats exposed to malathion alone. Therefore, it seems that the renal antioxidant status was matched with the results of the renal biochemical analysis (creatinine levels). This means that malathion induced its renal toxic effect via its interference with the renal antioxidant condition via the generation of free radicals (oxidative stress). 

Our results are in accordance with Alp and his colleagues, who demonstrated that malathion significantly increased MDA levels in the renal tissue, but curcumin could significantly reduce these values. GSH levels were dramatically decreased by malathion, while curcumin raised them by counteracting the impact of malathion in the kidney tissue [28]. In the same line, malathion administration was associated with an oxidative alternation that was measured by the lipoperoxidation process and MDA production in both renal and hepatic tissues [1]; however, curcumin therapy reduced the levels of MDA in the tissues and inhibited glutathione peroxidase activity, which mitigated the oxidative stress caused by malathion [7]. Treatment with curcumin restored the plasma TAS and reversed the depletion of erythrocyte GSH in fructose-fed rats, thereby augmenting the antioxidant defense system. It also inhibited the lipid peroxidation product, MDA, and the TOS/TAS ratio [29]. 

In the current work, Hematoxylin- and Eosin-stained sections showed the injurious effect of malathion toxicity on kidney tissue. These findings agreed with the outcomes of [1,30,31]. The malathion and curcumin-treated group showed more re-establishment of the typical structure compared with the malathion group. Some glomeruli appeared normal with normal Bowman’s capsules and others showed dilated capsules. Most tubules appeared normal. There was minimal interstitial tissue inflammation with mild hydropic degeneration in some tubules, in agreement with [32].

In the present study, the Sirius red stained section of the kidney of malathion-receiving rats revealed thicker collagen fibers around glomeruli, interstitial tissue, and renal tubules versus the control group; however, the curcumin and malathion-treated group showed thinner collagen fibers around the tubules and in the interstitium compared with the malathion-receiving group, consistent with the findings demonstrated by [33]. Curcumin exerts its antifibrotic action by reducing the extracellular matrix [34]; inducing apoptosis in myofibroblasts [35]; modulating inflammatory responses [36]; attenuating oxidative stress [37]; and inhibiting the NF-kβ signaling pathway [38].

In the current study, malathion significantly upregulated the expression of NFκβ in the kidney tissue and curcumin significantly ameliorated this elevation. This results in agreement with [39,40]; moreover, the same changes in the expression of TNF-α and curcumin reversed these changes, a finding similarly demonstrated by [41,42]. 

TNF-α is a cytokine that stimulates inflammation through tissue necrosis, apoptosis, cell proliferation, and innate and acquired immunity [43]. TNF levels in the serum were markedly elevated by malathion intoxication [44]. There were elevated IL-1, IL-6, and TNF-α levels, and afterward increased NF-KB levels in the renal tissues because of malathion administration. Activated TNF-α and induction of the NF-κβ signaling pathway can promote the translocation of NF-KB from the cytoplasm to the nucleus. By inhibiting NF-κβ, blocking its translocation to the nucleus, and preventing the gene’s expression of inflammatory cytokines, curcumin may delay the initiation of inflammation [45].

Candelario-Jalil et al. (2007) reported that the hepatoprotective effect of curcumin is related to its potent antioxidant activity and ROS-scavenging properties [46]. The ability of resveratrol and curcumin to overcome oxidative stress and decrease ROS production was reflected in the expressions of TGF-b1 and TGF-b2 in liver tissue. These findings showed the ability of polyphenolic compounds to inhibit the expression of TGF-b [47]. 

In the current work, the Nrf2 and HO-1-stained section of the malathion group revealed a negative reaction; on the other hand, curcumin rats revealed a strong positive reaction and significantly increased area% of positive immunoreactivity compared with the malathion group, and the same outcomes were documented by [42].

Nrf2 is a key regulator of cell survival that regulates antioxidant defenses, anti-inflammatory detoxification, and cell protection from oxidative stress and DNA damage [48]. Nrf2, which is the primary antioxidant system regulator, regulates cellular destiny by affecting cell division, proliferation, and apoptosis [49]. Antioxidant enzyme defense systems (including superoxide dismutase, reduced glutathione, and heme oxygenase 1) are directly regulated by Nrf2 [50]. Furthermore, it has been proved that curcumin inhibits the ROS-producing enzyme NADPH oxidase and activates endogenous antioxidant enzymes including HO-1 and SOD to prevent tumor invasiveness [51].

According to Lasram et al. (2014), malathion-induced liver inflammation was linked to decreased expression of the Nrf2 and HO-1 genes, which indicated the activation of inflammatory pathways, apoptosis, and fibrosis [24]. Nrf2 suppresses the oxidative stress-mediated activation of NF-κB by lowering intracellular ROS levels [52]. It also inhibits IκB-α proteasomal degradation and NF-κB nuclear translocation [53]. Keap1 suppression, control of Nrf2 expression and its target, and amplification of Nrf2 nuclear translocation may all contribute to the induction of Nrf2 signaling by curcumin [54]. The same pathway of oxidative stress, inflammation, and apoptosis was confirmed in many studies as in the Methotrexate-induced testicular injury [55] or Methotrexate-induced hepatic injury [56].

Attenuating the breakdown of IκB-α and up-regulation of Nrf2 causes an increase in cellular HO-1 levels [38]. Curcumin induced the expression of HO-1 in renal fibroblasts and human renal tubular cells [57]. HO-1 protein expression was increased by curcumin in a dose-dependent manner. Additionally, the anti-apoptotic action of curcumin was partially related to the activation of HO-1 [58,59,60]. Nrf2 activation resulted in a reduction in the production of COX-2 and Inos [61]. A concurrent increase in HO-1 and NQO1 content was discovered to be associated with the increased expression of COX-2 and iNOS and TNF-α and IL-6. This finding indicates that the Nrf2/HO-1 axis plays a role in inflammation [62].

Caspase-3 plays a crucial role in both intrinsic and extrinsic receptor-mediated apoptotic pathways [63]. In agreement with Sun et al. (2016), the current work revealed that curcumin significantly reduced the increased expression of Caspase-3 in the immunohistochemical stained sections resulting from malathion intoxication [64]. Malathion causes cellular stress, setting off a chain of events that eventually results in apoptosis [65]. Curcumin has an anti-apoptotic action. This can be explained by curcumin’s capacity to combat the lipid peroxidation and antioxidant depletion that trigger the apoptotic pathways [66].

The apoptosis of renal tubular cells was also decreased by curcumin administration [64,67] through a reduction in Caspase-3 activity in a dose-dependent manner [64]. According to Topcu-Tarladacalisir et al. (2016), renewal of the antioxidant system and lowered lipid peroxidation in rat kidneys are due to curcumin’s nephroprotective properties [67]. 

It has been demonstrated that curcumin possesses renal-protective properties [68]. Curcumin is an antioxidant and lipid peroxidation inhibitor [60,69,70], and it is known to have anti-inflammatory and anti-proliferative properties [71]. Additionally, it makes a defense against the harmful effects of various environmental contaminants and pesticides [72]. Curcumin is known for its diverse range of biological activities, including its ability to modulate various signaling pathways. One of the key mechanisms through which curcumin exerts its effects is by activating the Nrf2-ARE (Nuclear factor erythroid 2-related factor 2—Antioxidant Response Element) pathway. This pathway plays a central role in cellular defense against oxidative stress and inflammation by regulating the expression of various antioxidant and detoxification enzymes. The term “scavenger” is often used to describe compounds that directly neutralize and eliminate free radicals or reactive oxygen species. Curcumin does have some antioxidant properties but its primary mode of action is through the Nrf2-ARE pathway, which enhances the cellular defense mechanisms against oxidative stress. It can upregulate the production of endogenous antioxidants and enzymes, rather than directly scavenging free radicals like vitamins C or E. So, when curcumin is referred to as a “scavenger” in the context of the Nrf2-ARE axis, it is likely an oversimplification. It should be recognized for its broader role in promoting cellular resilience and protection against oxidative damage through the activation of this pathway in various tissues.

## 5. Conclusions

The current study reported that curcumin protects kidney tissue against malathion-induced nephrotoxicity. Additionally, this seemed to result from its anti-inflammatory, anti-apoptotic, and antioxidant capabilities. Curcumin administration restored the balance between oxidative stress and antioxidant activity through reduced TOS, OSI, and MDA, and increased TAC and GSH. Additionally, curcumin inhibits apoptosis (downregulates Caspase-3) and modulates markers of inflammation (NF-κβ/TNF-α, Nrf2, and HO-1) resulting from malathion intoxication.

## Figures and Tables

**Table 1:** Mean serum creatinine between studied groups.

	Group 1 Controls	Group 2 Curcumin	Group 3 Malathion	Group 4 Curcumin + Malathion	Test of Significance between Groups	Test of Significance between Groups and Control
Creatinine (mg/dl)	0.635 ± 0.017	0.51 ± 0.09	1.42 ± 0.34	0.973 ± 0.12	*p*1 = 0.001 * p2 = 0.001 * p3 = 0.001 *	*p*4 = 0.262 *p*5 = 0.001 * p6 = 0.005 *
Serum KIM-1 pg/mL.	653 ± 103.6	615 ± 96.7	2100 ± 258.2	1031 ± 173.1	*p*1 = 0.0001 * p2 = 0.0218 * p3 = 0.0001 *	*p*4 = 0.988 *p*5 = 0.001 * p6 = 0.037 *

Parameters labeled as mean ± SD; Test used: One-way ANOVA test; *statistically significant if *p* < 0.05. *p*1: the significance between curcumin and malathion groups; *p*2: the significance between curcumin and curcumin + malathion groups; *p*3: the significance between malathion and curcumin + malathion groups; *p*4: the significance between curcumin and control groups; *p*5: the significance between malathion and control groups; *p*6: the significance between curcumin + malathion and control groups.

**Table 2:** Effects of curcumin on oxidant and anti-oxidative status of normal and malathion-treated rats.

Tissue (Kidney)	Controls	Curcumin	Malathion	Curcumin + Malathion	Test of Significance between Groups	Test of Significance between Groups with Control
MDA (nmol/g.tissue)	5.61 ± 0.017	4.77 ± 0.43	11.05 ± 1.32	7.70 ± 1.29	*p*1 < 0.001 * p2 < 0.001 * p3 < 0.001 *	*p*4 = 0.142 *p*5 < 0.001 * p6 = 0.001 *
GSH (mmol/g.tissue)	2.10 ± 0.02	2.14 ± 0.19	0.603 ± 0.14	1.35 ± 0.22	*p*1 < 0.001 * p2 < 0.001 * p3 < 0.001 *	*p*4 = 0.678 *p*5 < 0.001 * p6 < 0.001 *
TOS (μmolH_2_O_2_ Eq./L)	22.5 ± 8.3	16.8 ± 10.8	52.7 ± 18.7	19.7 ±12.7	*p*1 < 0.001 * p2 < 0.001 * p3 < 0.001 *	*p*4 = 0.01 *p*5 < 0.001 * p6 = 0.001 *
TAC (μmol Trolox Eq t/l)	12.5 ± 3.7	17.9 ± 3.3	5.7 ± 2.9	18.2± 5.3	*p*1 < 0.001 * p2 < 0.001 * p3 < 0.001 *	*p*4 = 0.001 *p*5 < 0.001 * p6 = 0.001 *
OSI (H_2_O_2_/Trolox)	15.9 ± 5.3	11.3± 3.1	35.8 ± 7.3	21.9 ± 4.3	*p*1 < 0.001 * p2 < 0.001 * p3 < 0.001 *	*p*4 = 0.01 *p*5 < 0.001 * p6 = 0.001 *

Mean kidney tissue of MDA, GSH, TOS, TAC, and OSI were identified between studied groups. Parameters labeled as mean ± SD; Test used: One-way ANOVA test; * statistically significant if *p* < 0.05. *p*1: the significance between curcumin and malathion groups; *p*2: the significance between curcumin and curcumin + malathion groups; *p*3: the significance between malathion and curcumin + malathion groups; *p*4: the significance between curcumin and control groups; *p*5: the significance between malathion and control groups; *p*6: the significance between curcumin + malathion and control groups.

## Data Availability

The data presented in this study are available on request from the corresponding author. Data is not publicly available due to privacy.
